# Primary endocrine resistance of ER+ breast cancer with *ESR1* mutations interrogated by droplet digital PCR

**DOI:** 10.1038/s41523-022-00424-y

**Published:** 2022-05-02

**Authors:** Sung Gwe Ahn, Soong June Bae, Yoonjung Kim, Jung Hwan Ji, Chihhao Chu, Dooreh Kim, Janghee Lee, Yoon Jin Cha, Kyung-A Lee, Joon Jeong

**Affiliations:** 1 Department of Surgery, Gangnam Severance Hospital, Yonsei University College of Medicine, Seoul, Republic of Korea; 2 Institute for Breast Cancer Precision Medicine, Yonsei University College of Medicine, Seoul, Republic of Korea; 3 Department of Laboratory Medicine, Gangnam Severance Hospital, Yonsei University College of Medicine, Seoul, Republic of Korea; 4 Department of Surgery, Seoul St Mary’s Hospital, College of Medicine, The Catholic University of Seoul, Seoul, Republic of Korea; 5 Department of Surgery, Sacred Heart Hospital, Hallym University, Dongtan, Republic of Korea; 6 Department of Pathology, Gangnam Severance Hospital, Yonsei University College of Medicine, Seoul, Republic of Korea

**Keywords:** Breast cancer, Cancer genomics, Predictive markers

## Abstract

We investigated the patterns of recurrence and primary endocrine resistance according to estrogen receptor (ER) alpha gene (*ESR1)* mutations, as assessed by digital droplet (dd) PCR, in patients with non-metastatic ER+ breast cancer. We collected 121 formalin-fixed paraffin-embedded (FFPE) surgical specimens from ER+ breast cancer patients who had relapsed after surgery. Genomic DNA was extracted from the FFPE samples and *ESR1* mutations were evaluated using ddPCR. *ESR1* mutations were detected in 9 (7.4%) of 121 primary breast cancer specimens. The median recurrence-free interval and overall survival were significantly lower in patients with *ESR1* mutations than in those without. Of the patients treated with ET (*N* = 98), eight had *ESR1* mutations. Of these, six (75.0%) had primary endocrine resistance and two (25.0%) had secondary endocrine resistance. By contrast, only 22 of 90 (24.4%) patients without *ESR1* mutations had primary endocrine resistance. A multivariable model showed that an *ESR1* mutation is a significant risk factor for primary endocrine resistance. Our findings provide clinical evidence that the presence of rare *ESR1* mutant clones identified by ddPCR in primary tumors is associated with primary endocrine resistance in an adjuvant setting.

## Introduction

Mutations in the estrogen receptor (ER) alpha gene, *ESR1*, were first described in cell lines, when mutations in the ligand-binding domain (LBD), including Y537S and E380Q, were shown to constitutively activate the receptor^1^. Experiments with breast cancer cells harboring mutations in the LBD-encoding region of the *ESR1* gene have shown that mutant cells require a higher anti-estrogen drug concentration and that they proliferate in an estradiol-independent manner through constitutive activation of the ER pathway^1^. However, previous large-scale studies, such as The Cancer Genome Atlas project, have found that *ESR1* mutations are rarely detected in primary breast tumors (0.5% in 962 samples)^2^.

With the introduction of next-generation sequencing (NGS) technology in genomic research, *ESR1* mutations have been re-analyzed in samples from metastatic ER+ breast cancer. A series of studies has demonstrated that the incidence of *ESR1* mutations is as high as 11–55% in metastatic tumors samples from patients who previously underwent aromatase-inhibitor (AI) treatment^3–7^. Furthermore, using a hybridization capture-based NGS assay, known as the Memorial Sloan Kettering-Integrated Mutation Profiling of Actionable Cancer Targets (MSK-IMPACT) assay^8^, *ESR1* mutations have been detected in 3.5% (11 of 313) of primary breast cancer and 13.6% (84 of 616) of metastatic tumor samples^9^. These studies have collectively shown that *ESR1* mutations present rarely in primary treatment-naive ER+ breast cancer, whereas they are highly prevalent in metastatic tumors, suggesting that these mutations may potentially arise from rare clones of primary tumors through clonal selection against endocrine therapy (ET)^10–13^.

Despite the scarcity of *ESR1* mutations in primary ER+ breast cancer, several lines of evidence suggest that *ESR1*-mutated clones may be identified in primary tumors by droplet digital PCR (ddPCR)^14–16^. The rates of *ESR1* mutation detection are 2.6% to 12.0% in primary cancer when using ddPCR^14–16^. In ddPCR, template DNA is partitioned into approximately 20,000 droplets in a single reaction well and is then amplified within individual droplets. Therefore, this highly sensitive method has the capacity of providing accurate quantification without external references and is considered to be a useful tool to detect rare mutant alleles^17–19^. However, the clinical outcomes of patients with *ESR1-*mutated primary breast cancer are not well understood.

In this study, we sought to detect *ESR1* mutations using ddPCR in non-metastatic ER+ breast cancer. Moreover, we hypothesized that breast cancers harboring an *ESR1* mutation may show a different recurrence pattern compared to those with wild-type *ESR1*. We further addressed the relationship between the presence of an *ESR1* mutation and primary endocrine resistance in patients receiving adjuvant ET.

## Results

### Baseline characteristics and ESR1 mutations

**Fig. 1:**
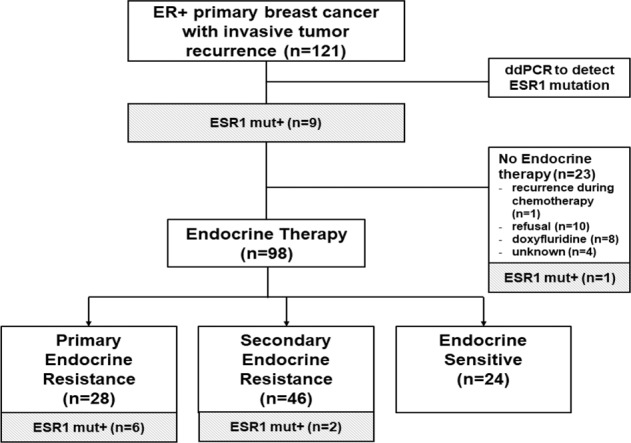
Consort diagram. The definitions of primary endocrine resistance, secondary endocrine resistance, and endocrine sensitivity followed the 5th International Consensus Conference for Advanced Breast Cancer guidelines and are provided in the Patients and methods section.

A total of 121 patients with recurrences were included in the study (Fig. 1). The median age at surgery for all patients was 45 years (range, 23–77 years). Among the 121 patients, 36 (29.8%) had stage I, 53 (43.8%) had stage II, and 32 (26.4%) had stage III breast cancer. Adjuvant chemotherapy was administered to 97 (80.8%) patients. In all, 98 (81.0%) patients received adjuvant ET, including tamoxifen (*N* = 59) and aromatase inhibitors (AIs, *N* = 39), whereas upfront anti-estrogen therapy was not administered to 23 (19.0%) patients (Supplementary Table 1).

**Fig. 2:**
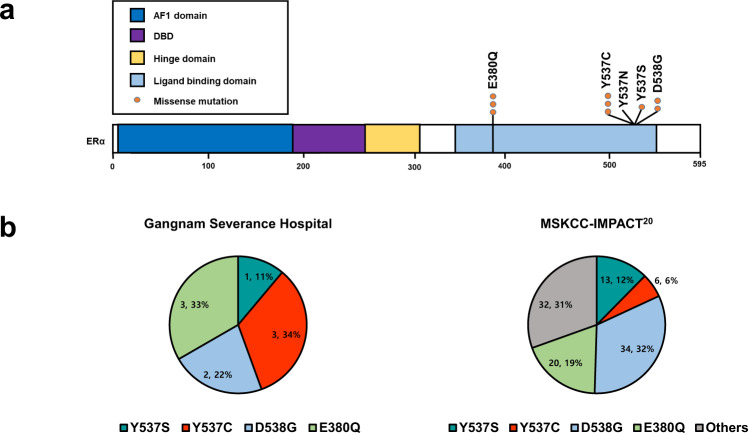
*ESR1* missense mutation in nine patients. **a** Locations and frequencies. **b** Distributions of ESR1 missense mutation sites: comparison the Gangnam Severance Hospital with the MSKCC-IMPACT series^20^.

*ESR1* mutations (E380Q, Y537C, Y537N, Y537S, and D538G) were detected by ddPCR. *ESR1* mutations were detected in 9 (7.4%, 95% Wald asymptotic confidence interval (CI) 2.8–12.1%) out of 121 primary breast cancer specimens (Fig. 2A). Y537C and E380Q mutations were found in three patients (33%), D538G mutation was found in two patients (22%), and Y537S mutation was found in one patient (11%). No Y537N *ESR1* mutations were detected. The median number of mutant allele copies was 2 (range, 2–587), and the median mutant allele fraction was 0.32% (0.01–8.37). The distribution of *ESR1* mutations in our cohort compared to that in the MSKCC-IMPACT series^20^ is illustrated in Fig. 2B.

When clinical and pathologic characteristics were compared based on the presence of *ESR1* mutations, the *ESR1*-mutation group had a higher T stage than the wild-type *ESR1* group (Supplementary Table 1). Other factors did not differ between the two groups.

The sites of the first recurrence are summarized in Supplementary Table 2. The most common site of the first tumor relapse was the bone (33.9%), followed by the lungs (24.0%) and distant lymph nodes (17.4%). There were no differences in the metastatic sites based on the *ESR1* mutations.

### Survival according to ESR1 mutation occurrence

**Fig. 3:**
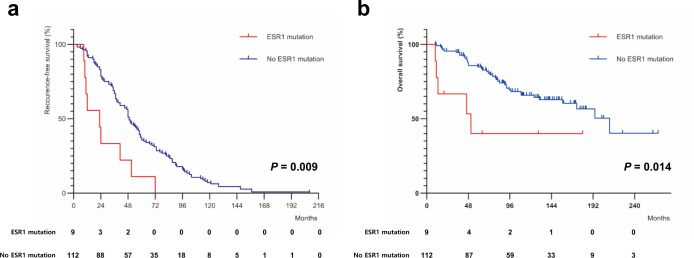
Kaplan-Meier survival plots according to *ESR1* mutation. **a** Recurrence-free interval. **b** Overall survival.

Recurrence-free interval (RFI) was defined as the time from the date of breast cancer surgery to the time of the first breast cancer recurrence, including locoregional and distant recurrences. Overall survival (OS) was defined as the time from the date of breast cancer surgery to the date of death from any cause or the last censored follow-up. The median follow-up time for the study population was 140 months (95% CI, 126–154 months). Since we selected patients with tumor recurrence, we compared the median RFI and OS using the Mann-Whitney U test. The median RFI was significantly lower in patients with an *ESR1* mutation than in those without an *ESR1* mutation (23.0 versus 49.0 months; *p* = 0.009). The median OS was 51 months in the *ESR1*-mutant group versus 211 months in the *ESR1*-wild-type group (*p* = 0.014). Survival plots for RFI and OS are presented in Fig. 3.

We analyzed whether ET type affected RFI stratified by *ESR1* mutation. The median RFI was not significantly different according to ET (tamoxifen vs. AIs), both in the *ESR1* mutation group (17.0 vs. 18.0 months; *p* > 0.999) and non-*ESR1* mutation group (48.0 vs. 58.0 months; *p* = 0.551). The survival plots are shown in Supplementary Fig. 1.

### ESR1 mutation and primary endocrine resistance

**Fig. 4:**
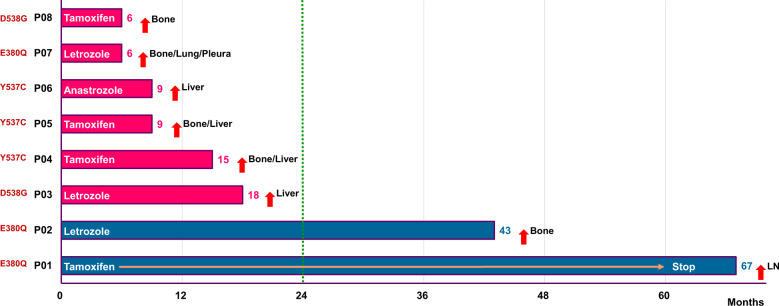
Recurrence types according to responsiveness to adjuvant endocrine therapy in eight patients with *ESR1* mutations who received adjuvant endocrine therapy. Among eight patients, six patients belonged to primary endocrine resistance, and two patients belonged to secondary endocrine resistance.

**Fig. 5:**
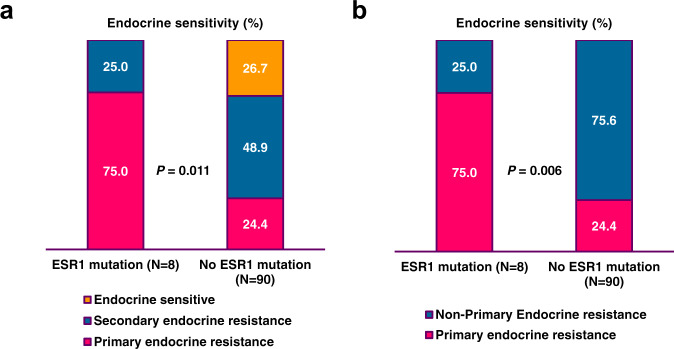
The relationship between *ESR1* mutation and recurrence type according to responsiveness to adjuvant endocrine therapy. **a** Primary endocrine resistance, secondary endocrine resistance, and endocrine sensitivity (*p* = 0.011; chi-square test) **b** Primary endocrine resistance versus non-primary endocrine resistance (*p* = 0.006; Fisher’s exact test).

Next, to assess the influence of *ESR1* mutations on primary endocrine resistance, we excluded patients without adjuvant ET (*N* = 23). The reasons for patients not undergoing ET are shown in Fig. 1. We classified the patients receiving adjuvant ET (*N* = 98) into three groups: (i) primary endocrine resistance (*n* = 28), defined as relapse during the first 2 years of adjuvant ET, (ii) secondary endocrine resistance (*N* = 46), defined as relapse during adjuvant ET, and (iii) endocrine sensitivity (*N* = 41), defined as not belonging to primary or secondary endocrine resistance^21^. The clinical and pathological characteristics of the three groups are presented in Table 1. Twenty-eight patients (28.6%) had primary endocrine resistance. Out of the eight patients with *ESR1* mutation, six (75.0%) had primary endocrine resistance and two (25.0%) had secondary endocrine resistance (Fig. 4). None of the endocrine sensitivity group had *ESR1* mutations detected. In contrast, only 22 out of 90 (24.4%) patients without an *ESR1* mutation had primary endocrine resistance, whereas 52 (48.9%) and 16 (26.7%) patients had secondary endocrine resistance and endocrine sensitivity, respectively (Fig. 5A, B).

**Table 1:** Baseline characteristics according to endocrine resistance in patients who received adjuvant endocrine therapy.

	Primary endocrine resistance (*N* = 28)	Secondary endocrine resistance (*N* = 46)	Endocrine Sensitive (*N* = 24)	Total (*N* = 98)	*P* value
Age (median, range)	45 (28–77)	44 (30–74)	48 (23–75)	45 (23–77)	0.973
Histologic type					0.789^a^
IDC	23 (82.1%)	42 (91.3%)	21 (87.5%)	86 (87.8%)	
ILC	2 (7.1%)	2 (4.3%)	1 (4.2%)	5 (5.1%)	
Others	3 (10.7%)	2 (4.3%)	2 (8.3%)	7 (7.1%)	
HG^b^					0.493^a^
1 or 2	21 (84.0%)	37 (86.0%)	21 (95.5%)	79 (87.8%)	
3	4 (16.0%)	6 (14.0%)	1 (4.5%)	11 (12.2%)	
LVI^b^					0.316
No	14 (66.7%)	27 (73.0%)	12 (92.3%)	53 (74.6%)	
Yes	7 (33.3%)	10 (27.0%)	1 (7.7%)	18 (25.4%)	
T stage					0.011^a^
1	8 (28.6%)	23 (50.0%)	13 (54.2%)	44 (44.9%)	
2	15 (53.6%)	23 (50.0%)	11 (45.8%)	49 (50.0%)	
3	5 (17.9%)	0	0	5 (5.1%)	
N stage					0.336^a^
0	11 (39.3%)	20 (43.5%)	11 (45.8%)	42 (42.9%)	
1	8 (28.6%)	16 (34.8%)	9 (37.5%)	33 (33.7%)	
2	3 (10.7%)	4 (8.7%)	4 (16.7%)	11 (11.2%)	
3	6 (21.4%)	6 (13.0%)	0	12 (12.2%)	
Stage					0.424
1	6 (21.4%)	14 (30.4%)	10 (41.7%)	30 (30.6%)	
2	12 (42.9%)	22 (47.8%)	10 (41.7%)	44 (44.9%)	
3	10 (35.7%)	10 (21.7%)	4 (16.7%)	24 (24.5%)	
Breast surgery					0.083
BCS	6 (21.4%)	18 (39.1%)	12 (50.0%)	36 (36.7%)	
TM	22 (78.6%)	28 (60.9%)	12 (50.0%)	62 (63.3%)	
Axillary surgery					0.142
SLNB	9 (26.5%)	8 (17.4%)	9 (37.5%)	26 (26.5%)	
ALND	19 (67.9%)	38 (82.6%)	15 (62.5%)	72 (73.5%)	
Adjuvant chemotherapy					0.426
No	4 (14.3%)	10 (21.7%)	7 (29.2%)	21 (21.4%)	
Yes	24 (85.7%)	36 (78.3%)	17 (70.8%)	77 (78.6%)	
Adjuvant endocrine					0.466
Tamoxifen	17 (60.7%)	30 (65.2%)	12 (50.0%)	59 (60.2%)	
AI	11 (39.3%)	16 (34.8%)	12 (50.0%)	39 (39.8%)	
Adjuvant radiotherapy					0.402
No	8 (28.6%)	20 (43.5%)	8 (33.3%)	36 (36.7%)	
Yes	20 (71.4%)	26 (56.5%)	16 (66.7%)	62 (63.3%)	

*IDC* invasive ductal carcinoma, *ILC* invasive lobular carcinoma, *HG* histologic grade, *LVI* lymphovascular invasion, *BCS* breast-conserving surgery, *TM* total mastectomy, *SLNB* sentinel lymph node biopsy, *ALND* axillary lymph node dissection, *AI* aromatase inhibitor.

^a^*P* values were obtained using Fisher’s exact test.

^b^Missing values.

In comparison with other clinical characteristics, T stage was higher in the group with primary endocrine resistance than in the groups with secondary endocrine resistance or endocrine sensitivity (Table 1). Accordingly, the group with primary resistance was more likely to receive a total mastectomy.

**Fig. 6:**
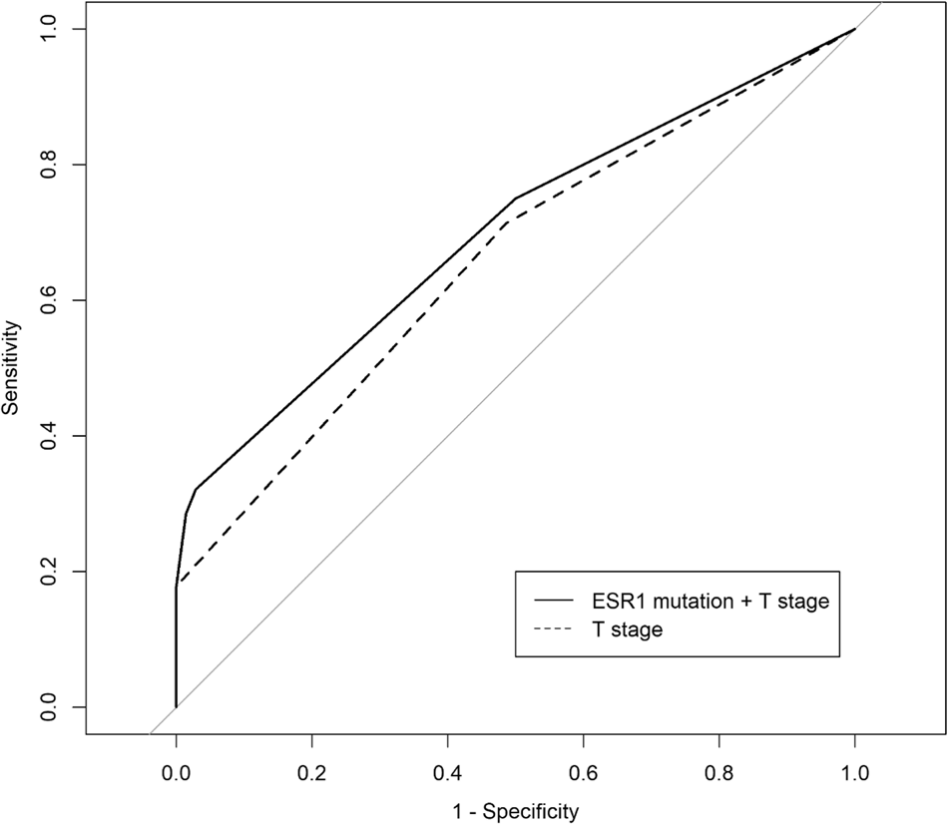
The areas under the curve (AUC) of two models. The AUC of the model with an *ESR1* mutation and T stage was numerically higher than that of the model with T stage alone. The AUCs of the two models were 0.698 (95% CI, 0.583–0.812) and 0.658 (95% CI, 0.545–0.770), respectively. There was no statistical difference when using the DeLong method.

To construct a multivariate model for primary endocrine resistance, univariate binary logistic regression analyses were first performed. *ESR1* mutation and T stage were found to be significant in these analyses (Table 2). The binary multivariate model demonstrated that an *ESR1* mutation was a significant factor for primary endocrine resistance, independent of T stage. The odds ratio of an *ESR1* mutation was 8.334 (95% CI, 1.524–45.561; Table 2), and the area under the curve (AUC) of the model consisting of an *ESR1* mutation and T stage was 0.698 (95% CI, 0.583–0.812; Fig. 6). The AUC of this model was numerically higher than that of the model with T stage alone (AUC, 0.658; 95% CI, 0.545–0.770). Within the subgroup (*N* = 74) nested by excluding endocrine-sensitive patients, an *ESR1* mutation was the only significant risk factor for primary endocrine resistance (Supplementary Table 3).

**Table 2:** Univariate and multivariate analysis of primary endocrine resistance in patients who received endocrine therapy.

	Univariable		Multivariable	
Variable	OR (95% CI)	*P* value	OR (95% CI)	*P* value
Age	0.999 (0.962–1.038)	0.976		
Histologic type				
IDC	Ref.			
Others	1.479 (0.704–3.104)	0.301		
HG				
1 or 2	Ref.			
3	1.578 (0.419–5.944)	0.500		
T stage				
1	Ref.		Ref.	
2 or 3	2.647 (1.029–6.807)	0.043	2.419 (0.907–6.453)	0.078
N stage				
Negative	Ref.			
Positive	1.228 (0.503–3.001)	0.652		
Adjuvant chemotherapy				
No	Ref.			
Yes	1.925 (0.585–6.333)	0.281		
Adjuvant radiotherapy				
No	Ref.			
Yes	1.667 (0.645–4.306)	0.292		
*ESR1* mutation				
No	Ref.		Ref.	
Yes	9.273 (1.744–49.305)	0.009	8.334 (1.524–45.561)	0.014

*OR* odds ratio, *CI* confidence interval, *IDC* invasive ductal carcinoma, *HG* histologic grade, *LVI* lymphovascular invasion.

## Discussion

Using ddPCR, we detected rare *ESR1* mutant clones in 9 of 121 (7.4%) primary ER+ breast cancer patients with relapse after surgery. Furthermore, we correlated the presence of an *ESR1* mutation with survival outcomes and found that the presence of ER+ treatment-naive tumors bearing an *ESR1* mutation was associated with primary endocrine resistance, despite their clonal rarity. This is the first report to provide clinical evidence that an *ESR1* mutation interrogated by ddPCR is linked with primary resistance to adjuvant ET in ER+ breast cancer.

Our ddPCR-based *ESR1* mutation detection rate was consistent with the rates reported in previous studies. These earlier studies identified *ESR1* mutant clones in 2.6% (7 of 270)^16^, 7.0% (3 of 43)^14^, and 12.0% (for Y537N)^15^ of primary cancers, respectively. As the study by Takeshita et al. included non-ER+ breast cancer^16^, it can be assumed that *ESR1* mutant clones may be present in more than 5% of primary ER+ breast cancers.

We detected a low *ESR1* mutation allele frequency, ranging from 0.01 to 8.37%, in primary ER+ breast cancer. This is similar to the findings of Wang et al., who reported *ESR1* mutant allele frequencies of 0.07 to 0.2% in ER+ primary breast cancer^14^. Due to the small number of cases with an *ESR1* mutation, we could not determine whether the mutant allele fraction was correlated with type of endocrine resistance. More data with a larger sample size are required to address this issue.

When we compared the distribution of *ESR1* LBD mutation sites between our cohort and the MSKCC-IMPACT series mainly consisting of ER+ metastatic breast cancer (Fig. 2B), Y537S (33%) and Y537C (33%) were observed most frequently in our cohort, while E380Q was observed at the highest frequency (32%) in the external cohort. Because the MSKCC-IMPACT used the NGS technique, they found additional *ESR1* mutations outside the LBD, including frame shifts or indels outside ESR1 LBD, with low frequency (*N* ≤ 2)^20^. To determine whether there is a difference in the *ESR1* mutation site between primary and metastatic breast cancer, further research is required.

Robust preclinical and clinical data suggest that *ESR1* mutations are associated with resistance to ET^5–7,10,13,15,22,23^. Mutations in the LBD-encoding region of the *ESR1* gene alter the structure of the ER protein, leading to ligand-independent activity^7,23^ and the recruitment of coactivators, such as SRC-1 and SRC-3^5,22^, which confer endocrine resistance. We investigated whether the presence of mutant *ESR1* in primary tumors affects endocrine resistance. Based on recent guidelines for the classification of endocrine resistance, we found that ER+ breast cancer patients bearing an *ESR1* mutation at surgery may have primary endocrine resistance. None of the eight subjects with an *ESR1* mutation showed a relapse pattern consistent with endocrine sensitivity.

If novel therapeutics that effectively eradicate mutant *ESR1* are employed clinically, the upfront use of the agents in an adjuvant setting has the potential to reduce failure of endocrine treatment. In the SoFEA (Study of Faslodex Versus Exemestane with or without Arimidex) trial, which was conducted in ER+ metastatic breast cancer, fulvestrant was shown to improve progression-free survival compared with exemestane in patients with an *ESR1* mutation, as detected in plasma samples by ddPCR^13^. Furthermore, novel selective estrogen receptor degraders (SERDs), which potentiate the degradation of mutant ER, have been under development and evaluated in clinical trials by several pharmaceutical companies^24^. For instance, an in vitro study showed that cancer cells with Y537S mutant *ESR1* are resistant to fulvestrant but sensitive to potent SERDs, such as AZD9496^20^.

Because we retrospectively identified and included only recurring patients with available primary surgical samples, our study has an inherent limitation of selection bias. Considering breast cancer recurrences continued to occur steadily after the end of ET^25^, the proportion of primary or secondary endocrine resistance was relatively high, at approximately 75%. This bias may have affected the ESR1 mutation rate and its subsequent prognostic impact. Therefore, our findings should be interpreted with caution. In addition, the lack of inclusion of non-recurring patients prevents formal assessment of primary *ESR1* mutations as biomarkers to guide ET. Further studies in a large prospective cohort with a sufficient follow-up period, including patients without relapse, are required to verify the findings.

Another limitation of our study was that we did not investigate *ESR1* mutations in serial tissue and blood samples from index patients. Analyses of matched primary and metastatic samples or serial plasma samples may elucidate how rare *ESR1*-mutant clones arise in primary tumors and become metastatic through dissemination in the bloodstream, in accordance with clinical tumor progression. However, metastatic tissues or blood samples were not available for these analyses. In future studies, the assessment of *ESR1* mutations in primary tumors and serial plasma samples will be essential for establishing therapies targeting mutant *ESR1*.

Furthermore, we could not assess the analytical sensitivity of this assay using synthetic templet or tumor genomic DNA which represented different levels of mutation abundance (%) for each mutation-specific probe in this study. However, according to Chu et al.^26^, the analytical sensitivity of *ESR1* mutation assay was reported at approximately 0.007%, and it was validated with clinical samples with 0.01% mutant alleles. Therefore, we determined 0.01% mutant alleles as a cut-off threshold and considered clinical samples with less than 0.01% of mutation fraction as an “*ESR*1 mutation-negative”.

Lastly, other *ESR1* mutations, such as K303R, L536P, and S463P^14,27–29^, were not included in our ddPCR panel. ddPCR assays targeting a larger number of mutations may affect the detection rate of *ESR1* mutations in primary cancer. In addition, our *ESR1* mutant detection rate should be interpreted with caution, considering that we only used tumor samples from subjects with relapse.

In conclusion, we showed that ddPCR detected rare clones with *ESR1* mutations in primary ER-positive cancer and we provided clinical evidence that the presence of rare *ESR1* mutant clones is associated with primary endocrine resistance in the adjuvant setting. We suggest that the detection of *ESR1* mutations in primary cancer by ddPCR may help predict failure during the early period of ET and help guide the early use of novel *ESR1*-mutant-targeting therapy.

## Methods

### Study population

Our study was approved by the Institutional Review Board of Gangnam Severance Hospital, Yonsei University, Seoul, Republic of Korea (IRB no. 3-2017-0349) and followed the Good Clinical Practice guidelines and the principles of the Declaration of Helsinki. The requirement for informed consent was waived due to the retrospective study design.

The medical records of 1667 patients with breast cancer who underwent breast surgery followed by adjuvant treatment at Gangnam Severance Hospital between January 1997 and December 2015 were reviewed. We identified 225 patients with primary non-metastatic ER+ breast cancer who experienced invasive tumor relapse after surgery. Formalin-fixed paraffin-embedded (FFPE) samples of primary tumors were available for ddPCR from 121 patients.

None of the patients had distant metastasis at the time of surgery. The available clinicopathologic data, including age; type of surgery; adjuvant treatment, including chemotherapy and ET; survival; ER status; HER2 status; histological type; histological grade; lymphovascular invasion status; and pathological stage. The consort diagram for the study population is displayed in Fig. 1. In the study population, 23 patients did not receive adjuvant ET due to the patients’ refusal (Fig. 1).

### Patient’s classification according to endocrine resistance

According to the 5th International Consensus Conference for Advanced Breast Cancer guidelines, we classified the 98 patients treated with adjuvant ET into the following three groups: (i) primary endocrine resistance, (ii) secondary endocrine resistance, and (iii) endocrine sensitivity^21^. Primary endocrine resistance was defined as relapse during the first two years of adjuvant ET. Secondary endocrine resistance was defined as relapse while on adjuvant ET, but after the first two years, or relapse within 12 months of completing adjuvant ET. The other patients were classified as endocrine sensitive.

### Droplet digital PCR

We collected 121 FFPE surgical specimens from patients with ER+/HER2− non-metastatic breast cancer. Representative tumor areas were identified, out of which at least three 10-μm-thick sections from the same FFPE samples were obtained, deparaffinized, and macrodissected. Genomic DNA was extracted using the QIAamp FFPE Tissue Kit (Qiagen, Venlo, The Netherlands) according to the manufacturer’s protocol. Digital PCR reactions were performed using a QX200 Droplet Digital PCR System and custom ddPCR assays (Bio-Rad Laboratories, Hercules, CA, USA). We detected E380Q, Y537C, Y537N, Y537S, and D538G mutations in the *ESR1* gene using probes targeting mutant and wild-type sequences, as previously described by Chu et al. and Jeselsohn et al.^26,30^. The primer and probe sequences are shown in Supplementary Table 4. The 20 μL PCR mix was composed of 10 μL of Bio-Rad ddPCR Supermix, 2 μL of each amplification primer/probe mix, and 8 μL of template DNA. Droplets then underwent the following thermal cycling protocol: one cycle of 95 °C for 10 min, followed by 40 cycles of 95 °C for 30 s and 46 °C (for E380Q) or 65 °C (for Y537 and D538G) for 1 min, followed by one cycle of 98 °C for 10 min. Results were analyzed using QuantaSoft v.1.7.2 software (Bio-Rad) and expressed as a percentage or fractional abundance of mutant DNA alleles compared to total DNA alleles.

When 20 non-tumorous samples were tested, 1 droplet/reaction was detected in 3 samples using E380Q mutation probe, and in 1 sample each using D538G and Y537N mutation probes. No positive droplets were detected in the results of the remaining mutation probes. Based on this, we set the limit of blank (LOB) at 0.857 copies/reaction (the highest value among LOB of mutation probes), and ddPCR result with a value of less than 2 positive droplets was reported as “not detected”.

### Statistical analysis

Categorical values were compared by chi-square or Fisher’s exact tests. The RFI was measured as the period from the date of breast cancer surgery to the first breast cancer recurrence, including locoregional and distant recurrences. OS was defined as the period from the date of breast cancer surgery to death from any cause or the last censored day. The medians of survival outcomes were compared using a Mann–Whitney *U* test because we only included patients with tumor recurrence. Binary logistic regression analysis was performed to identify independent factors associated with primary endocrine resistance. We determined the AUC of the multivariable model using a receiver operating characteristic curve with the DeLong method^31^. Variables with *p* < 0.05, in univariate analysis, were included in the multivariate analysis. All analyses were performed using IBM SPSS Statistics for Windows 23.0 (IBM Corp., Armonk, NY, USA) and SAS (version 9.3, SAS Inc., Cary, NC, USA). Statistical significance was defined as *p* < 0.05.

### Reporting summary

Further information on research design is available in the Nature Research Reporting Summary linked to this article.

## Supplementary information

Supplementary information

Reporting Summary

## Data Availability

We identified the distribution of ESR1 mutation in next-generation sequencing MSK-IMPACT data from the previous study^20^. The raw data supporting the conclusions of this article will be made available by the authors, without undue reservation.
