# Author Correction: Primary endocrine resistance of ER+ breast cancer with *ESR1* mutations interrogated by droplet digital PCR

**DOI:** 10.1038/s41523-022-00436-8

**Published:** 2022-05-18

**Authors:** Sung Gwe Ahn, Soong June Bae, Yoonjung Kim, Jung Hwan Ji, Chihhao Chu, Dooreh Kim, Janghee Lee, Yoon Jin Cha, Kyung-A Lee, Joon Jeong

**Affiliations:** 1 Department of Surgery, Gangnam Severance Hospital, Yonsei University College of Medicine, Seoul, Republic of Korea; 2 Institute for Breast Cancer Precision Medicine, Yonsei University College of Medicine, Seoul, Republic of Korea; 3 Department of Laboratory Medicine, Gangnam Severance Hospital, Yonsei University College of Medicine, Seoul, Republic of Korea; 4 Department of Surgery, Seoul St Mary’s Hospital, College of Medicine, The Catholic University of Seoul, Seoul, Republic of Korea; 5 Department of Surgery, Sacred Heart Hospital, Hallym University, Dongtan, Republic of Korea; 6 Department of Pathology, Gangnam Severance Hospital, Yonsei University College of Medicine, Seoul, Republic of Korea

**Keywords:** Breast cancer, Cancer genomics, Predictive markers

Correction to: *npj Breast Cancer* 10.1038/s41523-022-00424-y, published online 02 May 2022

In this article the funding from ‘This study was supported by Alvogen Korea.’ was omitted in the Acknowledgement. The original article has been corrected.

