# Supplementary material for: Primary endocrine resistance of ER+ breast cancer with ESR1 mutations interrogated by droplet digital PCR
Source: NPJ Breast Cancer. 2022 May 2;8:58. doi: 10.1038/s41523-022-00424-y (PMC9061813; doi:10.1038/s41523-022-00424-y)
Supplement: Supplementary file 1 — Supplementary information [file 41523_2022_424_MOESM1_ESM.pdf]

Supplementary information

Supplementary Table 1. Baseline characteristics according to *ESR1* mutation in all patients

	ESR1 no mutation (*N*=112)	ESR1 mutation (*N*=9)	Total (*N*=121)	*P*-value
Age (median, range)	45 (23-77)	53 (31-65)	45 (23-77)	0.554
Histologic type				0.279[a]
IDC	100 (89.3%)	7 (77.8%)	107 (88.4%)	
ILC	6 (5.4%)	1 (11.1%)	7 (5.8%)	
Others	6 (5.4%)	1 (11.1%)	7 (5.8%)	
HG[b]				>0.999[a]
1 or 2	87 (84.5%)	7 (87.5%)	94 (84.7%)	
3	16 (15.5%)	1 (12.5%)	17 (15.3%)	
LVI[b]				>0.999[a]
no	61 (77.2%)	6 (75.0%)	67 (77.0%)	
yes	18 (22.8%)	2 (25.0%)	20 (23.0%)	
T stage				0.015[a]
1	51 (45.5%)	2 (22.2%)	53 (43.8%)	
2	56 (50.0%)	4 (44.4%)	60 (49.6%)	
3	5 (4.5%)	3 (33.3%)	8 (6.6%)	
N stage				0.084[a]
0	49 (43.8%)	1 (11.1%)	50 (41.3%)	
1	36 (32.1%)	4 (44.4%)	40 (33.1%)	
2	14 (12.5%)	1 (11.1%)	15 (12.4%)	
3	13 (11.6%)	3 (33.3%)	16 (13.2%)	
Stage				0.158[a]
1	35 (31.3%)	1 (11.1%)	36 (29.8%)	
2	50 (44.6%)	3 (33.3%)	53 (43.8%)	
3	27 (24.1%)	5 (55.6%)	32 (26.4%)	
Breast surgery				0.156[a]
BCS	42 (37.5%)	1 (11.1%)	43 (35.5%)	
TM	70 (62.5%)	8 (88.9%)	78 (64.5%)	
Axillary surgery				0.445[a]
SLNB	30 (26.8%)	1 (11.1%)	31 (25.6%)	
ALND	82 (73.2%)	8 (88.9%)	90 (74.4%)	
Adjuvant chemotherapy				>0.999[a]
no	22 (19.8%)	1 (11.1%)	23 (19.2%)	
yes	89 (80.2%)	8 (88.9%)	97 (80.8%)	
Adjuvant endocrine				0.731[a]
no	22 (19.6%)	1 (11.1%)	23 (19.0%)	
Tamoxifen	55 (49.1%)	4 (44.4%)	59 (48.8%)	
AI	35 (31.3%)	4 (44.4%)	39 (32.2%)	
Adjuvant radiotherapy				0.296[a]
no	51 (45.5%)	2 (22.2%)	53 (43.8%)	
yes	61 (54.5%)	7 (77.8%)	68 (56.2%)	

IDC invasive ductal carcinoma, ILC invasive lobular carcinoma, HG histologic grade, LVI lymphovascular invasion, BCS breast conserving surgery, TM total mastectomy, SLNB sentinel lymph node biopsy, ALND axillary lymph node dissection,

AI aromatase inhibitor.

[a]*P*-values were obtained using Fisher's exact test.

[b]Missing values

Supplementary Table 2. Organ involvement in the first recurrence

	ESR1 no mutation (*N*=112)	ESR1 mutation (*N*=9)	Total (*N*=121)	*P*-value[a]
Bone	37 (33.0%)	4 (44.4%)	41 (33.9%)	0.486
Lung	26 (23.2%)	3 (33.3%)	29 (24.0%)	0.446
Distant lymph node	20 (17.9%)	1 (4.8%)	21 (17.4%)	>0.999
Ipsilateral breast or chest wall	20 (17.9%)	0	20 (16.5%)	0.353
Liver	16 (14.3%)	3 (33.3%)	19 (15.7%)	0.149
Pleura	9 (8.0%)	2 (22.2%)	11 (9.1%)	0.190
Ipsilateral axillary lymph node	7 (6.3%)	0	7 (5.8%)	>0.999
Brain	2 (1.8%)	0	2 (1.7%)	>0.999
Skin	2 (1.8%)	0	2 (1.7%)	>0.999
Ovary	2 (1.8%)	0	2 (1.7%)	>0.999

[a]*P*-values were obtained using Fisher's exact test.

Supplementary Table 3. Univariate analysis of primary endocrine resistance in the primary and secondary endocrine resistance groups

Variable	OR (95% CI)	*P*-value
Age	1.001 (0.960-1.044)	0.954
Histologic type		
IDC	Ref.	
Others	1.681 (0.705-4.005)	0.241
T stage		
1	Ref.	
2 or 3	2.500 (0.917-6.817)	0.073
N stage		
negative	Ref.	
positive	1.189 (0.457-3.094)	0.723
Adjuvant chemotherapy		
no	Ref.	
yes	1.667 (0.468-5.931)	0.281
Adjuvant radiotherapy		
no	Ref.	
yes	1.923 (0.703-5.259)	0.292
ESR1 mutation		
no	Ref.	
yes	6.000 (1.118-32.198)	0.009

OR odds ratio, CI confidence interval, IDC invasive ductal carcinoma, HG histologic grade, LVI lymphovascular invasion.

Supplementary Table 4. Primers and probes used in this study

Target	ddPCR Primers/probes	Sequence	Reference
E380Q	Forward Primer	TGGATTTGACCCTCCATGATCAG	[1]
	Reverse Primer	AGACGAGACCAATCATCAGGATCT	
	WT Probe	CCACCTTCTAGAATGTG	
	Mutant Probe	CCACCTTCTACAATGTG	
Y537S	Forward Primer	GCATGAAGTGCAAGAACGTG	[2]
	Reverse Primer	AAGTGGCTTTGGTCCGTCT	
	WT Probe	TCTATGACCTGCTGCTGGAGATGCT	
	Mutant Probe	TCTCTGACCTGCTGCTGGAGATGCT	
Y537C	Forward Primer	GCATGAAGTGCAAGAACGTG	[2]
	Reverse Primer	AAGTGGCTTTGGTCCGTCT	
	WT Probe	TCTATGACCTGCTGCTGGAGATGCT	
	Mutant Probe	TCTGTGACCTGCTGCTGGAGATGCT	
Y537N	Forward Primer	GCATGAAGTGCAAGAACGTG	[2]
	Reverse Primer	AAGTGGCTTTGGTCCGTCT	
	WT Probe	TCTATGACCTGCTGCTGGAGATGCT	
	Mutant Probe	TCAATGACCTGCTGCTGGAGATGCT	
D538G	Forward Primer	GCATGAAGTGCAAGAACGTG	[2]
	Reverse Primer	AAGTGGCTTTGGTCCGTCT	
	WT Probe	TCTATGACCTGCTGCTGGAGATGCT	
	Mutant Probe	TCTATGGCCTGCTGCTGGAGATGCT	

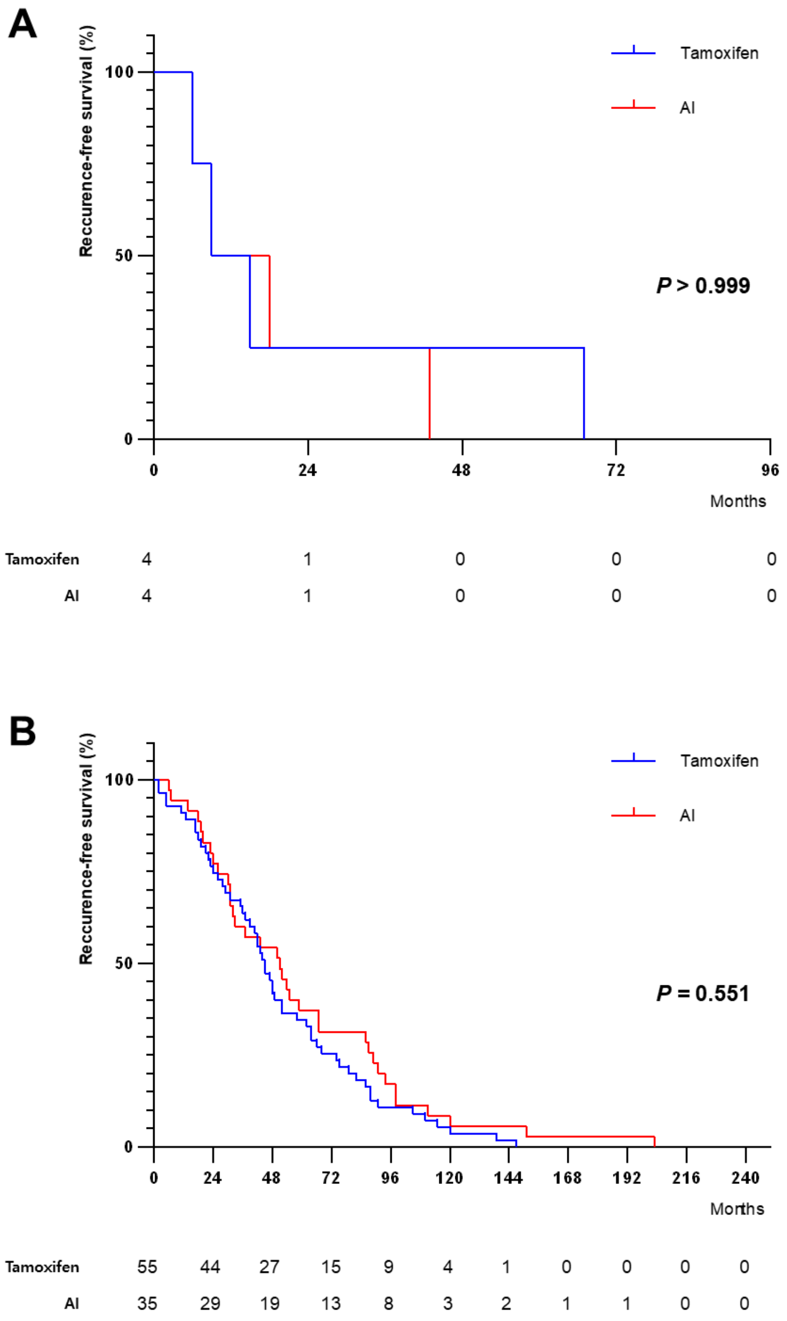

Supplementary Figure 1. Recurrence-free interval according to endocrine therapy (A) in patients with ESR1 mutation, and (B) in patients without ESR1 mutation
